# ‘A disastrous blow’: psychiatric risk, social indicators and medical authority in abortion reform in post-war Britain

**DOI:** 10.1136/medhum-2018-011561

**Published:** 2019-05-30

**Authors:** Sarah Crook

**Affiliations:** Department of History, Swansea University, Swansea, UK

**Keywords:** abortion, post-war, psychiatry, legal reform, medicine

## Abstract

The Second World War lent impetus to the creation of new models and explanatory frameworks of risk, encouraging a closer reading of the relationship between individual psychiatric disorder and social disarray. This article interrogates how conceptions of psychiatric risk were animated in debates around abortion reform to forge new connections between social conditions and psychiatric vulnerability in post-war Britain. Drawing upon the arguments that played out between medical practitioners, I suggest that abortion reform, culminating in the 1967 Abortion Act, was both a response to and a stimulus for new ideas about the interaction between social aetiologies and medical pathologies; indeed, it became a site in which the medical and social domains were recognised as mutually constitutive. Positioned in a landscape in which medical professionals were seeking to assert their authority and to defend their areas of practice, abortion reform offered new opportunities for medical professionals to intervene in the social sphere under the guise of risk to women’s mental health. The debate in medical journals around the status of issues that were seen to bridge the social and the medical were entangled with increasing anxiety about patient agency and responsibility. These concerns were further underscored as conversations about psychiatric risk extended towards considerations of the potential impact on women’s existing families, bringing domestic conditions and the perceived psychosocial importance of family life into relief within medical journals. This article, then, argues that conceptions of psychiatric risk, as refracted through the creation of new synapses connecting the social and the medical domains, were critical to medical debates over abortion reform in post-war Britain.

## Introduction

Four years before the Abortion Act 1967, psychiatrist Myre Sim declared in the *British Medical Journal* (*BMJ*) that ‘there are no psychiatric grounds for the termination of pregnancy’, for abortion was a socioeconomic problem, with the psychiatrist merely providing a means to circumvent restrictive legislation.1 Sim was quickly rebuked: another psychiatrist, Roger Tredgold, pointed to the legal consensus that ‘psychiatric grounds *do* exist’.2 The psychiatrist, Tredgold claimed, could only assess the likelihood of mental disorder based on individual circumstance, and socioeconomic factors were often too integral to this to ignore.3 Underlying the controversy was the question of the extent to which ostensibly social factors affected mental well-being and how the risk to mental health could be implicated in medical decisions.

The Abortion Act 1967, as originally enacted, decreed that abortion was lawful if two registered medical practitioners agreed that ‘the continuance of the pregnancy would involve risk to the life of the pregnant woman, or of injury to the physical or mental health of the pregnant woman, or any existing children of her family…In determining whether the continuance of a pregnancy would involve such risk of injury to health…account may be taken of the pregnant woman's actual or reasonably foreseeable environment’.4 Thus, the Abortion Act 1967 asserted risk as a critical metric in the decision-making process in abortion cases. Within the medical and legal landscape, ‘mental health’ became a means of acknowledging the environmental and social strains of unwanted childbearing. The Act therefore embodied a new understanding of the intersections and overlapping interests of social and medical reproductive care.

By 1970, three-quarters of all legal abortions in England and Wales were performed on psychiatric grounds, although one physician observed in a letter to *The Lancet* that ‘these grounds are usually those of psychological reaction to the environmental situation created by an unwanted pregnancy’.5 In a House of Lords debate in 1969, it was reported that 28 849 abortions were carried out under the Act in England and Wales between 27 April 1968 and 25 February 1969. Of these, 20 746 were conducted due to ‘risk to physical or mental health of woman’.6 Clearly, the threat to mental health constituted a significant point of access to medical terminations.

This article explores how the medical community navigated the relationship between social and environmental factors and medical categories in post-war Britain during debates about abortion reform. I argue that risk was a critical lexicon in the abortion debates of the 1960s. Put another way, I suggest that it was under the auspices of risk to women’s mental health that concerns about the personal implications and social contexts of unwanted pregnancies were made legitimate within the medical domain. Drawing on medical literature and legislative debates from the 1960s, this article is a contribution to the literature on the history of abortion reform in Britain. It is also in dialogue with scholarship that charts the emergence of risk as a concept that traversed social, legal and medical domains in the post-war period.

## Abortion and risk

This article, therefore, is in conversation with multidisciplinary scholarship that has recently emerged around the mechanisms and processes through which abortion was medicalised. Fran Amery, for example, has argued that the medicalisation of abortion was an ‘incomplete and fragmentary process’, engaging the state and the medical profession in a complex discussion about medical responsibility and sociomedicine.7 As Amery notes, the extension of the concepts of health and medicine through abortion debates was not welcomed ubiquitously; rather, they were ‘fraught with difficulty’ and ‘caused rifts between Parliament and the medical profession’.8 The medical profession, as Sheelagh McGuinness and Michael Thomson have shown, was also riven with internal disputes: they identify the relationship between the Royal Colleges as particularly important to the trajectory of abortion services.9 Abortion, McGuinness and Thomson argue, is ‘one boundary issue among many that helped to define medicine and, in so doing, the content of proper medical practice’.10 Risk, I contend, was one way that this professional boundary was defined. This article, then, addresses the history of the regulation of abortion and how this was shaped by the importance granted to the metric of risk. It demonstrates the way that medical risk became embedded in legislation and was articulated in and through debates about an issue that operated at the boundary of the social and the medical. Significantly, the relationship between legislation and medical expertise remains contested. As Ellie Lee has shown, the purported threats to women’s mental health posed by abortion continue to be deployed by those seeking to curtail women’s access to the reproductive procedure.11 The discourse of risk continues to be mobilised, too, by those seeking to liberalise abortion. Indeed, recent calls for abortion legislation to be liberalised have been made on the grounds that ‘legislation has to match the science’, arguing that the legislation should be brought ‘in line with modern medicine and legislation’.12 Arguments about medical abortion—in which two pills are taken—are now put forward in terms of the relative safety of this method, and the risks posed by forcing women to be administered these pills in a clinical setting, away from the potential comforts provided by their home, are brought to the fore.13 Changes to abortion legislation, then, remain framed within the discourse of risk. These discourses continue to challenge the clinical setting and appropriateness of the medical oversight of abortion.

The rise and origins of risk have been richly theorised, perhaps most notably by Ulrich Beck. Beck argued that as risks are dependent on choices, they are ‘*politically reflexive*’; that the sources of danger were not ‘ignorance but *knowledge*’.14 As this article demonstrates, the knowability of patients’ contexts was a critical issue in debates over abortion reform. As Robert Castel has pointed out, ‘new strategies dissolve the notion of a *subject* or a concrete individual, and put in its place a combination of *factors,* the factors of risk’.15 Put another way, a ‘risk does not arise from the presence of a particular danger embodied in a concrete individual or group. It is the effect of a combination of abstract *factors* which render more or less probable the occurrence of undesirable modes of behaviour’.16 Risk attained a particular importance in post-war Britain: Nikolas Rose has argued that while public health has been attentive to risk as a metric since the 19th century, ‘risk thinking’ assumes a particular significance and character in societies dominated by pharmaceutical modes of treatment.17 The global rise of pharmacological interventions in the post-war period has indeed been charted by historians.18 The late-20th century, Rose has concurred with Castel, oversaw a shift from the move from dangerousness to risk in psychiatric thinking.19 ‘The responsibilities of almost all psychiatric professionals have come to be redefined in terms of the assessment of risk’ Rose observes, and through the ‘generalisation of the criterion of risk’ mental health professionals ‘participate in the management of individuals’.20 In the case of abortion reform, of course, doctors oversaw the management of individual patients, and the criterion of risk was made official in legislation. Anthony Giddens observed that much contemporary politics is ‘now about managing risks—risks which do not originate in the political sphere, yet have to be politically managed’.21 Abortion reform legislation, passed through parliament, formalised medical management of the risk posed by unwanted pregnancies. Through the examination of the ways that risk was implicated in abortion reform, we can come to understand how psychiatric risk made the social visible in post-war Britain.

Ellie Lee has shown that the Abortion Act embodied contemporary understandings of a ‘set of interrelated social questions’ and that the medicalisation of abortion made the ‘assessment of the mental health of the woman… a significant feature of the operation of abortion law’. This, she suggests, allowed women access to legal abortion but inscribed their perceived mental vulnerability into law.22 I argue that the Act also articulated a connection between risk and mental health in post-war legislation. I contend that the process of legislative change around abortion in the 1960s acted as what Richard Ericson and Aaron Doyle have termed a ‘system’ by which ‘risks are given meaning and acted on’.23 As Ericson and Doyle contend, ‘Risk is called into being, made visible, and responded to through the rules, formats, and technologies available… The communication system makes risks real’.24 Around abortion, psychiatric risk was ‘called into being’ by the medical profession’s various organisations and the legislative framework of the British state; the dialogue between medical bodies and Parliament created a ‘communication system’ and made risk a critical category of analysis in abortion cases. These legislative and professional communication systems imbued psychiatric risk in abortion cases with meanings drawn from the perceived psychosocial significance of the family environment that were in circulation in the years preceding the passage of the Act.

## Wrecks and risks before the 1967 Abortion Act

Prior to the passage of the 1967 Abortion Act, the law governing abortion in England and Wales was principally drawn from sections 58 and 59 of the Offences Against the Person Act 1861. As Sally Sheldon has observed, the Act was ‘widely criticised as anachronistic and archaic’ and did not articulate any exceptions for therapeutic abortions or contain any time limits.25 Time limits were subsequently introduced by the Infant Life (Preservation) Act 1929, although as Sheldon notes, this was introduced not to address abortion but rather to address the legal loophole around killing a child in the midst of being born. This Act proscribed the destruction of a child ‘capable of being born alive’ and decreed that this capacity began at 28 weeks’ gestation. Significantly, the Act also decreed that ‘no person shall be found guilty of an offence under this section unless it is proved that the act which caused the death of the child was not done in good faith for the purpose only of preserving the life of the mother’.26 As Stephen Brooke has said, however, this definition of life did not explicitly allow for the consideration of economic, social or psychological indications.27 This did not mean, of course, that mental health indications were not taken into account in practice; indeed, John Keown has demonstrated that doctors defended an expansive definition of health in the 1920s.28 Nonetheless, by the late 1930s, there were varying opinions among doctors as to when abortion was permissible.29

The opportunity to challenge and clarify the law arose in 1938. Overseen by Mr Justice Macnaghten, the case of *Rex v. Bourne* concerned an abortion carried out by the gynaecologist Dr Aleck Bourne.30 His patient was a 14-year-old girl who had been raped by a group of soldiers; as a result of the termination, Bourne was charged under the Offences Against the Person Act 1861.31 The case hinged on whether a distinction could be drawn between preserving health and saving life.32 Bourne’s defense was that he performed the termination due to the inter-relationship between ‘danger to life and danger to health’ and that the girl’s mental health would be endangered by completing the pregnancy.33 Bourne was found not guilty and the case set a precedent in allowing for a broader interpretation lawfulness of abortion under existing legislation.34

The judgement was not considered entirely satisfactory, however, Bourne’s acquittal was rued in *The Lancet* as having ‘left the legal position—except for two welcome passages in the judge’s summing-up…only a little less obscure than before’.35 Justice Macnaghten digressed ‘from purely physical indications in order to give support to the view that termination is also lawful in those cases where the mental health of the mother is seriously threatened’.36

Discussion focused on Macnaghten’s use of non-medical language. Macnaghten stated that juries would be sympathetic to the doctor who operated under the belief that to continue the pregnancy would be to risk rendering the woman a ‘physical or mental wreck’.37 ‘If pregnancy is likely to make the woman a physical or mental wreck’ the judge concluded, ‘the jury is entitled to take the view that a doctor, who in the circumstances and led by his belief operates, is operating for the purpose of preserving the life of the mother’.38 Hailed by psychiatrist Montague Joyston-Bechal as a ‘typical example of the maturity of the English legal system’, the term ‘mental wreck’ was described as ‘picturesque rather than precise’, the ambiguity of which ‘creates both its strength and its weakness’.39 Joyston-Bechal continued,

‘wreck’ is sufficiently emotive a term to discourage any who might be tempted to co-operate in ridding a woman of the distress appropriate to a temporary embarrassment. Also, ‘wreck’, being ill defined, can embrace any number of individual variations of psychiatric sequelae to pregnancy. Many regard this ambiguity as a weakness, offering such little guidance that they are restrained from recommending termination unless the wreck is total—presumably derelict and unfloatable. Most of us take the view that the law can be interpreted more widely and that although we terminate to prevent the development of a serious and prolonged psychiatric disorder, this might not necessarily be permanent, or incapacitating.40

The term thus allowed for some clinical autonomy. Nonetheless, the phrase was a cause of consternation and was criticised for being ‘scarcely scientific’.41

The ambiguity of the Bourne judgement, perhaps unsurprisingly, created discrepancies how the phrase was interpreted in practice.42 Some doctors interpreted the Bourne judgement to mean that the *potential* for damaged mental health justified termination, while others sought evidence that it was a certainty.43 In 1964, R F Tredgold observed that psychiatrists had to come to two main judgements: first, would ‘the patient’s health break down irretrievably’ and second, ‘will she commit suicide, if pregnancy goes on?’.44 This standard, as Keown has shown, was not ubiquitous. Under the ‘Bourne’ judgement, psychiatrists had some leeway in implicating mental health in abortion judgements; the 1967 legislation provided explicit formal grounds for this. Psychiatrist James Arkle reflected in 1957 that Macnaghten’s phrase ‘mental wreck’ was important for psychiatrists in that it ‘makes it justifiable for him to recommend termination if he honestly believes that continuation of a pregnancy will make a woman a mental wreck. Lesser degrees of emotional upset are not enough, nor are social disturbances of any magnitude nor eugenic forebodings of any kind’.45 Against this limited interpretation of Macnaghten’s statement, the 1967 Act can be seen as a significant extension and expansion of the concept of risk in abortion cases.

## Rising expectations and cultures of risk control

First, though, it is necessary to set out why the concept of ‘risk’ attained such influence in post-war Britain. The period following the Second World War marked the recognition of an expansive definition of well-being, codified by the WHO in the late-1940s, when it defined health as ‘a state of complete physical, mental and social well-being’.46 This broad definition of health allowed doctors greater scope for medical intervention. Greater interventionalism was enabled by a culture of rising expectations of health in Britain after the creation of the National Health Service (NHS), for under the NHS illnesses could be managed. Moreover, the increased availability of contraception in the decades after the Second World War rendered childbearing and its attendant strains increasingly avoidable. The 1949 report of the Royal Commission on Population noted that women were no longer willing to tolerate excessive childbearing, instead preferring their ‘independent status’ and ‘wider interests’.47 Indeed, Dugald Baird commented in 1966 that ‘instead of fatalistically accepting a succession of unplanned pregnancies, the mother is now determined to have the number of children she wants and feels capable of caring for’.48 In 1971, it was suggested by Norman Todd, a consultant psychiatrist, that raised expectations might explain the increased demand for therapeutic abortion; ‘it may be that women are less able or willing to adapt to, or even tolerate, the burden of unwanted pregnancy as they were in the past’.49 For Bourne, defendant in the precedent-setting 1938 case, the relationship between unlimited childbearing and illness was clear: the strain of ‘repeated and unwanted pregnancies’ would render women ‘tired, lifeless and worn out’, and ‘fear, depression and fatigue’, would ‘exact its price in the form of physical symptoms’.50 By the mid-1960s, women were increasingly able to implement family planning measures. The introduction of the contraceptive pill on the NHS in 1961 signified a broader acknowledgement that family planning contributed to individual, familial and national well-being.

It was argued in 1966 that the women of the 1960s were ‘less timid, less furtive, more determined and more practical’ and were capable of demanding an abortion from a doctor under ‘reasonable conditions’.51 Baroness Summerskill contended in 1965 that abortion was ‘a matter in which the voice of women should be the deciding factor’, for it was their ‘human rights’ under consideration and the views of the Church should not be decisive for ‘it is not for celibate men to decide the fate of these women’.52 Unwanted motherhood, here, was not framed as a fate to be endured. Sociologist John Peel claimed that ‘a responsible attitude towards parenthood and a desire to protect the interests of an existing family will not be lightly sacrificed for an unplanned pregnancy’, a trend he identified as part of ‘the revolution of rising expectations’.53 The relationship between family planning and women’s expectations of their life cycle was clear: ‘when a woman has resumed work outside the home, after careful planning of her family, an unwanted pregnancy can be a disastrous blow’.54 In 1973, Raymond Illsley noted the connections between contraception and women’s assertiveness around abortion: ‘the more emphasis we place on family planning the more likely it is that patients themselves will claim the right to decide. The delicate interchange of hints and clues in the doctor–patient encounter… has already changed and will continue to change towards a more direct exchange of request and opinion’.55

As Alex Mold has argued, the 1960s and 1970s witnessed a shift towards a greater recognition of the agency of the patient.56 The culture of paternalism within which the patient submitted unquestioningly to the will and expertise of the physician was beginning to change: instead, the patient as a consumer was emerging.57 In the context of the 20th-century renegotiation of the doctor–patient relationship, the anxieties expressed by doctors regarding the accessibility of the legal framework highlighted the patient’s particular role as an active agent in seeking the abortion procedure. Drew Halfmann suggests that doctors objected to legal clauses that would ‘create categories of patients who were eligible by law for abortions, thus removing the necessity of doctors’ diagnoses and turning doctors into ‘mere technicians’; in Britain, he shows, this concern was principally about patient infringement on professional discretion.58

Abortion reform was seen to put clinical autonomy at risk through its creation of the knowledgeable and entitled female patient. As pointed out by S J Macintyre, from the Centre of Social Studies at the University of Aberdeen, the medical profession maintained ‘mystique’ and authority through the relative opacity and inaccessibility of the diagnostic and treatment criteria for most medical practices.59 In the case of abortion, however, legal reform rendered the criteria for treatment visible.60 Correspondence to the *British Medical Journal* suggested that gynaecologists were worried that they would ‘have women and their relatives “breathing down their necks” if the Bill provides a codified list of indications for termination’.61 Here the risk was that the law might facilitate ‘abortion on demand’, fundamentally reworking the power dynamic of the doctor–patient relationship.62

## Medical cultures, professional bodies and risk

There was a growing sense of optimism about the potential for interventions in mental health in mid-20th century Britain: the numbers of in-patient beds in psychiatric hospitals declined from 1954, new drugs became available and the 1962 Hospital Plan proposed the integration of psychiatric units into general hospitals.63 Mary Boyle has argued that the 1959 Mental Health Act had important implications for the place of mental health in abortion reform through its recognition of mental illness as equivalent to physical illness and through its affirmation of doctors as impartial, authoritative adjudicators.64 By the 1960s, Boyle contends, definitions of ‘psychological harm’ had become elastic enough to accommodate the arguments for abortion reform.65 This occurred against a backdrop of the development of the welfare state; a model of national organising that has been framed by David Garland as a ‘risk management state’.66 Indeed, risk has been argued to have been critical to the rationale of the British welfare state, constituting the state’s ‘response to the problem of handling the risks encountered in a typical life course’.67 The risk of ill health was targeted by the establishment of the NHS. It was within this broader transformation to the landscape of mental health that abortion reform occurred and within which medical bodies representing different medical specialisms sought to intervene.

From the early 1960s, medical organisations accepted that abortion reform was likely; what form this might take and how it would affect medical autonomy became primary concerns.68 As Keown has argued, professional autonomy was a major concern of the organisations that represented the specialisms of the medical profession.69 Michael Thomson has suggested that abortion was a boundary issue for the medical profession in the 20th century.70 Certainly in the period preceding the 1967 Act, medical bodies published a flurry of memoranda on abortion reform. The contribution of the British Medical Association (BMA) was particularly important.71 The BMA’s July 1966 report emphasised practitioner discretion, the exclusive right of the medical practitioner to undertake abortions and opposed non-medical grounds for termination.72 In so doing, it asserted its members as the sole legitimate arbitrators and necessitated the medicalisation of women’s options. The BMA suggested that a termination might be lawful if two medical practitioners agreed, and it granted that the likelihood of foetal deformity could be taken into account.73

The Royal Medico-Psychological Association published a memorandum on abortion reform in July 1966. It approved the inclusion of social, medical and psychiatric indications in the termination decision, as they contributed to the ‘promotion of health and the prevention of disease’, and suggested that in ‘addition to traditionally accepted medical and psychiatric criteria, all social circumstances should be taken into account’.74 The Association advised that the patient ‘must be viewed in the total context of the woman’s individual, family, social and life experience’.75 The report argued that in the case of a ‘severe chronic mental illness…there is a prima facie case for therapeutic abortion’.76 While it reflected that this should not automatically lead to termination, it commented that the children of ‘feckless and irresponsible’ parents, incapable of fulfilling their parental duties, were prone to being ‘unhappy and mentally disordered and are particularly prone to behave in an antisocial manner’; thus, ‘the likelihood of serious parental inadequacy of this sort does…constitute grounds for termination of pregnancy’.77

The Royal College of Obstetricians and Gynaecologists (RCOG) published the most conservative of the professional association reports.78 It suggested that the majority of gynaecologists opposed immediate reform of the abortion law. Moreover, it proclaimed change unnecessary: ‘we are unaware of any case in which a gynaecologist has refused to terminate pregnancy, when he considered it to be indicated on medical grounds, for fear of legal consequences’.79 The College suggested that psychiatric symptoms could be ‘exaggerated’.80 Furthermore, it was argued that suicide following a refused abortion was uncommon.81 Risk of suicide, it is worth noting, may have been underestimated due to coroners’ reluctance to record it as cause of death, instead recording it as due to the less stigmatised death by misadventure, accident or under an open verdict.82 It argued to intervene was to put women at greater risk of mental disorder:

There are few women, no matter how desperate they may be to be find themselves with an unwanted pregnancy, who do not have regrets at losing it. This fundamental reaction, governed by maternal instinct, is mollified if the woman realizes that abortion was essential to her life and health but if the indication for the termination of pregnancy was flimsy and fleeting she may suffer from a sense of guilt for the rest of her life.83

The BMA and the RCOG published a joint report on abortion reform in late 1966.84 The report was concerned that the bill introduced by Liberal MP David Steel contained too wide a social clause (stipulating that therapeutic abortion was permissible in the case of rape, or if the pregnancy posed a significant risk to the woman’s capacity to mother), creating scope for ‘abortion on demand’.85 ‘An excessive demand for abortion on social grounds’, the bodies noted, ‘would be unacceptable to the medical profession’.86 Instead, they suggested that the patient’s ‘total environment’, ‘actual or reasonably foreseeable’ could be considered.87 In lieu of social language, medical organisations proposed that the social and psychological environment be drawn into the medical framework.

The implications of this expansion of medical discretion were discussed earlier in the 1960s, during the consideration of Lord Silkin’s proposed abortion reform bill, which preceded Steel’s private members bill.88 Lord Silkin’s 1965 speech in the House of Lords invoked the uncertainty of doctors in interpreting the law as it stood, as well as the ‘public expense’ of treating illegally attempted abortions.89 Silkin emphasised that his Bill would facilitate doctor discretion and noted that it enabled responsiveness to the ‘health of a patient or the social conditions which make her unsuitable to assume the legal or moral responsibility of parenthood’.90 Responding to Lord Silkin’s proposal of a second reading of his Bill, Viscount Dilhorne agreed with the need for legislation, and reflected professional medical bodies’ concerns that taking the social implications of an unwanted pregnancy into account might require a ‘remarkable degree of prescience on the part of medical practitioners’ and cautioned that some of the provisions of the Bill, such as allowing abortion for pregnancies brought about by rape, might ask doctors to ‘undertake what they are not really well fitted to perform’.91 Instead, he claimed that ‘surely the test in such cases should be whether the continuation of a pregnancy is likely to cause serious injury to the mental or physical health of the woman or girl’.92 Although he supported reform, he cautioned that terminating a pregnancy was a serious choice and framed this within the discourse of risk: ‘I understand that it is wrong to suppose that the operation, even if performed in the early days of pregnancy, and when properly conducted, is entirely without risk. There is a risk of trauma, physical or mental, which may be serious and prolonged, and possibly irreversible; and there may be physical results as well’.93 The Lord Bishop of Southwark reiterated anxieties that the social frame did not accord with the qualifications of medical professionals, suggesting that it ‘places too heavy a responsibility on the medical practitioner’.94 Beyond this, he asked ‘Are we to assume that a degree in medicine gives to the holder of it such insights into sociological problems that he is competent to determine by himself, and without consulting anybody else, what are suitable and unsuitable social conditions?’95 The respondents to Silkin’s Bill asserted the importance of the medical profession, with Lord Stonham, the joint parliamentary undersecretary of state in the Home Office, making this connection explicit: ‘We must also attach special weight to the views of the medical profession. The proposed changes would impose considerable responsibilities on doctors, and we have to be sure that they are willing and able to carry them’.96

## Risk, stress and the sociomedical environment

In 1971, David Steel M P wrote of the increased recognition of the interdependence of ‘social conditions’ and ‘medical considerations’.97 He even suggested that the drafting of the Act ‘encouraged the concept of sociomedical care’.98 Sociomedical care contributed to the medicalisation of areas of life previously not under the auspices of the medical profession.99 One psychiatrist declared in 1966 that his profession was as interested in social context as social reformers due to the ‘inevitable influence of environment on mental health’.100 Writing 1 year before the passage of the 1967 Act, Anderson E W claimed that ‘no psychiatrist needs to be reminded of the importance of the social factor both in the aetiology and the prognosis of all mental illness regardless of its form. The social factor in effect weighs as heavily as the medical’.101 Here the social and the medical were considered distinct but complementary. The patient was socially situated within the clinical setting, and the physician was positioned to consider how social and environmental factors might impinge on the health of the individual patient.

That the Abortion Act drew the social into medical view under the guise of psychiatric risk was not uncontroversial. It was thought after the passage of the Act that the indications for termination had led to ‘widespread misconception’ that abortion could be secured on social grounds.102 One psychiatrist lamented that ‘psychiatric diagnosis is reputed to be soft, flexible and accommodating enough to be used to achieve whatever goals the diagnostician wishes to reach’.103 This demonstrated that ‘the label of threat to the mental health of the subject may be regarded as a convenient method of achieving abortion on demand under a different guise’.104 This was reiterated by an international report written by Raymond Illsley and Marion H Hall in the late 1970s, which noted ‘psychiatry became the Trojan horse by which liberal abortion was introduced into societies with restrictive laws but humane ideologies’.105 In Britain, the Trojan horse took the guise of the acknowledgement of the traffic between social and medical indications by the risk of psychiatric disorder. Illsley had noted this in 1973, observing that ‘Psychiatry had already proved, in many spheres, a discipline amenable to the pressure of changing social values and customs… But could one justify termination on psychiatric grounds for a young woman whose past history showed no signs of behavioural or personality disorder, and whose distress arose from the consequences of thoughtless and hedonistic acts or their stigmatisation by society?’. The answer to this was, he suggested, often in the affirmative: ‘the consequences were real enough for the patient whose welfare the physician accepted as part of his professional responsibility’.106 In 1966, Sir Dugald Baird, formerly Regius Professor of Midwifery at the University of Aberdeen, claimed that it was indefensible to exclude social factors, for this took little heed of ‘the effect of customs, tradition, education, the new status of women in society and a host of other factors which influence health, happiness and efficiency’.107

The concept of stress was one way that the social and the medical were bridged to articulate the risks posed by an unwanted pregnancy. As David Cantor and Edmund Ramsden have argued, by the mid-20th century stress had become one of the dominant lexicons through which anxieties over the nature of modernity could be expressed.108 The vocabulary of stress was deployed to account for an increasing number of psychological and physical reactions to life events, reframing the relationship between the external world, the body and the mind.109 The psychological gaze encroached further into public and private life as psychological experts encouraged the public to consider their personal experiences within the framework of stress.110 Mark Jackson has argued that the amelioration of stress ‘promised new therapeutic options’ for a society in the midst of cultural change.111 The stress discourse mapped onto social anxieties, and engaged increasingly with the psychological and the social, rather than the biological or hormonal.112 Baird noted that his study, carried out in Scotland, revealed that between the years of 1961 and 1963, the percentage of pregnancies terminated on surgical or medical grounds remained stable, but the percentage carried out for psychiatric reasons more than doubled.113 This, he explained, was due to the emergence of a ‘very important new group’, identified as ‘suffering from emotional and physical stress aggravated by adverse emotional and living conditions’.114 Rhodri Hayward has argued that stress was a ‘productive concept’, providing ‘a kind of conceptual glue which allowed individual failings…to be joined to broader transformations in society or the environment’.115 The concept of stress traversed socio-economic groups, allowing working-class and middle-class women to engage with the same need for abortion. Stress thus provided a legitimising terminology for the association between social causes of medical consequences.116 Some were provoked to ask if the pregnancy was a ‘final straw’ that ‘simply puts too much stress’ on the patient.117

An interest in the psychosocial aspects of abortion was reflected in the medical interest in adverse reactions to the procedure. In 1976, Raymond Illsley, Director of the Medical Research Council Medical Sociology Unit, and Marion Hall, Consultant in Obstetrics and Gynaecology at the Aberdeen Teaching Hospitals, published an extensive review of the psychosocial aspects of abortion in the *Bulletin of the World Health Organization*.118 In it, they acknowledged not only that attitudes to abortion were culturally contingent, but that women’s emotional and psychiatric responses to abortion were shaped by the societies from which they emerged. Indeed, they identified guilt over abortion as ‘traditionally induced as part of a traditional system of social control’ and argued that ‘in such circumstances, it is superfluous to ask whether patients will experience guilt—it is axiomatic that they will’.119 Roger Tredgold had similarly claimed in 1966 that the psychiatric aftermath of abortion was ‘to some extent affected by the attitude of the ward and especially of the gynaecologist and nurse’, for they might, from time to time, ‘vent their feelings on a patient whose story makes little appeal to their sympathy’.120

Although there was significant debate about the emotional sequelae, studies found that adverse psychiatric reactions to termination were rare.121 Peter Diggory, a gynaecologist, claimed that abortion ‘relieves the strain under which the woman was breaking, and if followed by adequate contraceptive advice…there may be little further need for psychiatric help’.122 Indeed, abortion might maintain a woman as a ‘useful member of the community’, which supported his view that abortion might sometimes be ‘merely a part of the psychiatric treatment’.123 This consideration of the potentially prophylactic effects of termination reflected a broader turn towards the significance of mothering in post-war Britain.

## Mothering and risk

Tolerance of ostensibly social reasons for abortion reform, although under the guise of psychiatric risk, was facilitated by post-war ideas of maternal responsibility for child psychosocial development. However, grounds for this were laid earlier in the 20th century; during the 1930s advocates of safe abortion had suggested that it would support the family unit and safeguard maternal health.124 By the post-war period, medical experts argued that a married woman seeking to limit family size would see ‘a striking improvement’ in her own health, that of her family, marital relations, and ‘a more congenial home atmosphere’, and indeed, the ‘removal of the constant threat of pregnancy allows the woman to be a better wife and mother’.125 Within this framework, abortion was not an emancipatory tool but a means of supporting the psychosocial role of the family. Effective family planning measures, including access to abortion, would reinforce rather than undermine the family and social aspirations.

The influence of John Bowlby’s attachment theory affirmed beliefs that the unwanted child faced and posed psychosocial challenges.126 It was wondered prior to reform what implication liberalisation would have for rates of ‘juvenile delinquency, alcoholism, mental deficiency, suicides, homicides, arrests’.127 This reveals some of the enduring influence of the eugenicist language that was deployed in social debates earlier in the century.128 There was some concern in the House of Lords that the social scope of abortion reform might facilitate ‘a certain amount of pseudo-eugenics’.129 Baroness Wootton of Abinger noted that ‘there is a real risk that, if we allow a social clause, we shall be allowing the medical profession to make judgments on considerations which are not medical but social…I am anxious that we should be absolutely clear of pseudo-eugenics and regard this Bill entirely from the point of view of the pregnant woman, her welfare and the welfare of the child she may be about to bear’.130

Nonetheless, it was considered by some doctors to be a social good to prevent the ‘spread’ of undesirable behaviours by facilitating safe abortion. In 1966, it was claimed by a senior doctor in psychological medicine that, ‘now that it is almost axiomatic that delinquency is associated with bad homes, it seems illogical to insist that an unwanted child shall be brought into the world, not only into bad physical circumstances but with a parent who will not love it’.131 In 1965, one member of the House of Lords said that access to medical abortion would prevent child rearing from threatening to ‘wear down the personality of the mother until she becomes just a drudge’; a childcare environment that would risk creating a ‘malformed or mentally defective child who has no real prospect of ever becoming a real human personality’.132 Ben Clements and Clive D Field have examined opinion polls and sample surveys to review trends in public attitudes towards abortion since the 1967 Act. They show that according to a 1964 National Opinion Polls (NOP) study, 49% of respondents rejected abortion arising from an inability to cope with any more children, with just 44% of respondents in favour. By 1967, these opinions had shifted: two NOP surveys found that 65% accepted abortion in these circumstances.133 It is worth noting that there was stronger consensus around the acceptability of abortion in situations in which women’s health was endangered. As Clements and Field note, in 1966, a Gallup poll found that 79% of respondents accepted abortion in such situations, a percentage that rose to 86 in 1967.134 In 1980, a MORI study found that 91% of respondents approved of access to abortion when the woman’s life was endangered.135 Interestingly, the British Social Attitudes survey used the language of endangering—the phrasing of the question was ‘The woman's health is seriously endangered by the pregnancy’—signalling the ways that the legal language of risk had elided with endangerment in public discourse.136

The family’s psychological significance to child mental health provided a legitimising frame for abortion reform. Madeleine Simms of the Abortion Law Reform Association suggested that personality development theories endorsed women’s right to make decisions over ending a pregnancy.137 Simms noted that ‘if a woman deeply resents the birth of an unwanted child or is incapable of mothering satisfactorily too many children, then the consequences in terms of mental health for that child and the rest of her family are grave’; indeed, Simms warned, ‘she may be laying the foundation of psychiatric illness in the next generation’.138 In Lord Silkin’s speech to the House of Lords, he noted the broad coalition of support for the principles underpinning his 1965 Bill for abortion reform, including the ‘National Council of Women, the Women’s Co-Operative Guild and other women’s organisations… the Magistrates Association and the Eugenics Society’.139 The interests of groups engaged with women’s social position and those concerned with apparently undesirable social behaviours met under the auspices of abortion reform. The post-war emphasis on the significance of ‘good enough mothering’ ran alongside an anxiety over the social effects of the poor family environment.

## Risk and the investigative medical professional

As Sheldon has observed, however, abortion reform was not wholly liberalising; instead, she contends, ‘women seeking termination were decriminalised in order to be pathologised’.140 Gendered constructions of the female patient retained significant sway in debates over abortion reform in the early 1960s. Sheldon has pointed to images of femininity that were drawn on in the debates: women as minors, as victims, and as mothers, and as irresponsible and untrustworthy.141 The construction of the untrustworthy female patient, however, had implications for the medical professional and their role in judging psychiatric risk.

The possibility of deception by female patients preoccupied some doctors. ‘How is the doctor to know that the patient…is not lying about the alleged misfortune which makes or will make the bearing of a child intolerable?’, one asked in the *BMJ*.142 Women were accused of telling ‘fictions’ including ‘heart-rending stories of brutal husbands or landlords or rape by mental defectives, even of risk of hereditary transmission of disease’ in order to secure an abortion.143 Here the female patient was not only configured as untrustworthy, but liable to use her knowledge of the legal grounds for abortion to her advantage. This, too, was associated with risk: risk requires the truth to be apparent, so probabilities can be evaluated and threats accounted for. The woman who wanted something—namely an abortion—could obscure the true metrics of risk by misleading a doctor. In 1965, a member of the House of Lords cautioned that pressure for abortion reform was not coming from the medical profession, and that that there was a concern that ‘doctors will be inundated with ladies whose contraceptives have not worked and who threatened to have nervous breakdowns unless doctor terminates the pregnancy.144

One correspondent to the *BMJ* suggested that nursing staff and mental welfare officers might be drawn on to make ‘suitable inquiries’.145 Within this rubric, patient authenticity was under investigation, expanding the responsibilities of the medical professional. Others were concerned that pregnant women were too volatile to make informed decisions; some correspondence to medical journals supported the idea that it was unkind to give women sole responsibility over abortion decisions, as pregnancy rendered women ‘emotional’ and their judgement ‘unsound’.146 One doctor reflected that women tended to ‘improvise’ their attitudes to abortion ‘only when already in a state of confusion and distress’.147 Others advised that women were liable to change their mind: ‘how many politicians have any first-hand experience of the often surprising as well as gratifying manner in which many women later become reconciled to an “unwanted” child and thank their medical attendant for refusing to consider abortion?’148 The debate over abortion thus revealed tensions over patient power in an era of rising expectations of health. There were clear anxieties that female patients, and their families, might seek to persuade doctors of their poor psychological health through deceptive means. That the informed patient might seek to avoid the hardships imposed by an unwanted pregnancy through utilising the tools of the medical profession was considered to have the potential to transform the doctor–patient dynamic. It suggested a new role for the physician in the social landscape as an arbitrator of risk but also required a renegotiation of power in the consulting room.

## Conclusion

This article has argued that abortion reform in the late 1960s supported a broader understanding of the interaction between the social and the medical domains in the medical imagination and that metrics of risk were critical to this. I suggested that raised expectations of health, well-being and the responsibilities of the family facilitated greater medical intervention into women’s reproductive lives. It was also enabled by the development of the NHS and the broader welfare state. Psychiatric risk emerged from abortion legislation as an expansive category through which women could access medical terminations for reasons informed by their social situation. In the 1970s, Irving Zola noted that medicine was encroaching into the social sphere and that in issues like abortion moral issues were displaced by a debate that focused on ‘the degree of sickness attached to the phenomenon in question or the extent of the health risk involved’.149 The question of extent of risk, as we have seen, preoccupied the medical profession in the years after the Bourne decision, leading to the 1967 Abortion Act. More broadly, as scholars have highlighted, the rise of the concept of risk has not been limited to abortion but has more generally come to play an increasingly significant role in parents’ lives.150

We have seen that those supporting and challenging the passage of abortion legislation in the 1960s invoked the views of medical bodies. This was not a straightforward assertion of the primacy of their expertise, however: abortion reform also facilitated a discussion about the boundaries of medical decision-making and required a negotiation of the concept of risk. In one way, at least, the risks posed by unwanted pregnancies were successfully addressed: within a decade of reform rates of emergency admission due to incomplete miscarriage and abortion had declined by two-thirds, indicating that the reform had significantly improved women’s access to safe family limitation.151 Beyond this, abortion reform had provoked a rearticulation of medical professionals’ roles at the intersections of the domestic, social and medical spheres. More recently, marking the 50 year anniversary of the 1967 Abortion Act, scholars have interrogated the extent of the authority that the Act granted to the medical profession, arguing that discussions with doctors who provide abortions highlight the importance they ascribe to women’s own authority and opinion in making this reproductive decision; moreover, as Lee, Sheldon and Macvarish show, ‘those doctors most involved in providing abortions place moral value on this work’.152 The attribution of authority to women is reflected in broader public support for women’s ‘right to choose’.153 As highlighted earlier in the article, demands are increasingly being made for the decriminalisation of abortion and for further liberalisation. These calls are grounded in a conviction that the regulation of abortion should be brought into line with other medical procedures but also deploy the discourse of safety. Clare Murphy of the British Pregnancy Advisory Service has argued that the current requirement for two doctors’ authorisation causes delays and has noted that ‘Abortion procedures today are safe and straightforward and do not need to be performed by doctors. However, the law currently denies nurses and midwives a larger role in the provision of care’.154 Thus, safety, antonymic to risk, continues to play a role in abortion debates and to feature in discussions about which groups of medical professionals play a role in abortion services. More broadly, the regulation of abortion in Britain reflects ‘the poor alignment between the ageing statutory framework and contemporary clinical understandings of best practice in abortion services’, Sally Sheldon has recently argued.155 As we can see, then, the relationship between risk and abortion, established in earlier conversations around legal reform, has contemporary resonance.

## Notes

1. Myre Sim (1963), “Abortion and the Psychiatrist.” *British Medical Journal,* 2, no. 5350 (July 1963), 145.

2. RF Tredgold (1963), “Abortion and the Psychiatrist,” *British Medical Journal* 2, no. 5358 (September 1963), 681. (Emphasis in original)

3. Ibid, 681.

4. Her Majesty's Stationery Office (2019), “Abortion Act 1967”. (London: HMSO, 2019).

5. J Crawford (1970), “Abortion Act,” *The Lancet*, 296, no. 7683 (November 1970), 1138.

6. HL Deb, 25 March 1969, vol. 300, cc 1135–1137, 1135.

7. Fran Amery (2014), “Social Questions, Medical Answers: Contesting British Abortion Law,” *Social Politics: International Studies in Gender, State & Society,* 21, no. 1 (March 2014): 28, https://doi.org/10.1093/sp/jxt003.

8. Ibid, 33.

9. Sheelagh McGuinness and Michael Thomson (2015), “Medicine and Abortion Law: Complicating the Reforming Profession,” *Medical Law Review*, 23, no. 2 (June 2015): 177–199.

10. Ibid, 198.

11. Ellie Lee (2003) , *“Abortion, Motherhood and Mental Health: The Medicalization of Reproduction in the United States and Britain* (New York: Aldine Transaction, 2003).

12. Katherine Cowles (2018), “Our Abortion Law is Archaic, Marked by Avoidable Cruelties,” *The Guardian,* 25 March, 2018, https://www.theguardian.com/commentisfree/2018/mar/25/abortion-law-outdated-women-have-right-to-dignity [Accessed 17 October 2018].

13. Ibid.

14. Ulrich Beck (1997) , *“Risk Society: Towards a New Modernity”* (London: Sage, 1997), 183.

15. Robert Castel (1991), “From Dangerousness to Risk,” in *The Foucault Effect: Studies in Governmentality,* eds. Graham Burchell, Colin Gordon and Peter Miller (Chicago: University of Chicago Press, 1991), 281.

16. Ibid, 287.

17. Nikolas Rose (2005), “In Search of Certainty: Risk Management in a Biological Age,” *Journal of Public Mental Health*, 4, no. 3 (2005): 14.

18. See, for example, Andrea Tone (2009), *The Age of Anxiety: A History of America’s Turbulent Affair with Tranquillisers* (New York: Basic Books, 2009).

19. Nikolas Rose (2002), “At Risk of Madness,” in *Embracing Risk: The Changing Culture of Insurance and Responsibility,* eds. Tom Baker and Jonathan Simon (London and Chicago: University of Chicago Press, 2002), 210–211.

20. Ibid, 216.

21. Anthony Giddens (1999), “Risk and Responsibility,” *The Modern Law Review*, 62, no. 1 (January 1999): 1–10.

22. Ellie Lee (2002), “Psychologising Abortion: Women’s ‘Mental Health’ and the Regulation of Abortion in Britain,” in *Well Women: the Gendered Nature of Healthcare Provision,* eds. Anne Morris and Susan Nott (Aldershot: Ashgate, 2002), 61.

23. Richard Ericson and Aaron Doyle (2003), “Risk and Morality,” in *Risk and Morality* (Toronto: Toronto University Press, 2003), 2.

24. Ibid, 2.

25. Sally Sheldon (1997) , *“Beyond Control: Medical Power and Abortion Law* (London and Chicago: Pluto Press, 1997), 14.

26. legislation.gov.uk (1929), “Infant Life (Preservation) Act, 1929. 19 &amp 20 Geo. 5 C.34”.

27. S Brooke (2001), “‘A New World for Women?’ Abortion Law Reform in Britain during the 1930s,” *The American Historical Review*, 106, no. 2 (April 2001): 432, https://doi.org/10.1086/ahr/106.2.431.

28. John Keown (1988) , *“Abortion, Doctors and the Law: Some Aspects of the Legal Regulation of Abortion in England from 1803 to 1982,”* (Cambridge: Cambridge University Press, 1988), 72–73.

29. The Lancet (1938), “A Charge of Illegal Abortion, Rex v. Bourne,” *The Lancet*, 232, no. 5995 (July 1938): 220.

30. RW Durand (1938), “Abortion: Medical Aspects of Rex V. Bourne,” *The Modern Law Review,* 2, no. 3 (December 1938): 236.

31. A.C.H.B (1941), “Criminal Law—Procurement of Miscarriage—Defence that Operation was to Save Mother’s life—Continuance of Pregnancy would Probably Bring about Physical and Mental Wreck of Mother,” *The Cambridge Law Journal,* 7, no. 2 (July 1941); 270, https://doi.org/10.1017/S0008197300125516.

32. The Lancet (1938), “Rex V. Bourne Again,” *The Lancet* 232, no. 6004 (September 1938): 731–732, https://doi.org/10.1016/S0140-6736(00)40966–9.

33. The Lancet (1938), “A Charge of Illegal Abortion, Rex v. Bourne,” *The Lancet,* 232, no. 5995 (July 1938): 220–226, 220.

34. W Lindesay Neustatter (1948), “*Modern Psychiatry in Practice*.” 2nd ed. (London: J&A Churchill, 1948), 243.

35. The Lancet (1938), “Rex V. Bourne,” *The Lancet,* 232, no. 5995 (July 1938): 201. https://doi.org/10.1016/S0140-6736(00)82499-X

36. James Arkle (1957), “Termination of Pregnancy on Psychiatric Grounds,” *British Medical Journal,* 1, no. 5018 (March 1957), 559.

37. Macnaghten, quoted in Arkle, “Termination,” 559.

38. Ibid.

39. MP Joyston-Bechal (1966), “The Problem of Pregnancy Termination on Psychiatric Grounds,” *Journal of the College of General Practitioners,* 12, no. 3 (November 1966): 306.

40. Ibid, 306.

41. RF Tredgold (1966), “The Psychiatrist’s View,” *Abortion in Britain: Proceedings of a Conference held by the Family Planning Association at the University of London Union on 22 April 1966* (London, 1966), 10–11.

42. W Lindesay Neustatter (1966), “Change of Outlook,” *Abortion in Britain: Proceedings of a Conference Held by the Family Planning Association at The University of London Union on 22 April 1966* (London, 1966), 22.

43. AA Baker (1967), “*Psychiatric Disorders in Obstetrics”* (Oxford: Blackwell, 1967), 127.

44. RF Tredgold (1964), “Psychiatric Indications for Termination of Pregnancy,” *The Lancet,* 284, no. 7372 (December 1964): 1252, https://doi.org/10.1016/S0140-6736(64)92732-1.

45. Arkle, “Termination,” 559.

46. The Constitution of the World Health Organization (1947), *WHO Chronicle,* 1947, 1–2.

47. Royal Commission (1949), “*Report of the Royal Commission on Population.”* Cmd 7695 (London: HMSO, 1949), 148.

48. Dugald Baird (1966), “Experience at Aberdeen,” *Abortion in Britain: Proceedings of a Conference Held by the Family Planning Association at the University of London Union on 22 April 1966”* (London, 1966), 16.

49. Norman A Todd (1971), “Psychiatric Experience of the Abortion Act (1967),” *British Journal of Psychiatry,* 119, no. 552 (1971): 494, doi:10.1192/bjp.119.552.489.

50. A Bourne (1959), “Discussion on Psychosomatic Gynaecology,” *Journal of Obstetrics and Gynaecology,* 66 (October 1959): 771.

51. R Woolf, “Changes,” *Abortion in Britain: Proceedings of a Conference Held by The Family Planning Association at the University of London Union on 22 April 1966* (London, 1966), 70.

52. Deb HL, 30 November 1965, vol. 270, cc. 1192.

53. J Peel (1966), “Attitudes in Britain,” in *Abortion in Britain: Proceedings of a Conference Held by the Family Planning Association at the University of London Union on 22 April 1966”* (London, 1966), 68.

54. Dugald Baird (1966), “Experience at Aberdeen,” 16.

55. Raymond Illsley (1973), “Foreword” in *Experience with Abortion: A Case Study of North-East Scotland*, ed. Gordon Horobin (Cambridge: Cambridge University Press, 1973), ix.

56. Alex Mold (2013), “Repositioning the Patient: Patient Organizations, Consumerism, and Autonomy in Britain during the 1960s and 1970s,” *Bulletin of The History of Medicine*, 87, no. 2 (July 2013): 225–249.

57. Ibid.

58. Drew Halfmann (2003), “Historical Priorities and the Responses of Doctors’ Associations to Abortion Reforms in Britain and the United States, 1960–1973,” *Social Problems,* 50, no.4 (November 2003): 570–577, https://doi.org/10.1525/sp.2003.50.4.567.

59. S J Macintyre (1973), “The Medical Profession,” 128.

60. Ibid, 128.

61. DP Cocks (1967), “Termination of Pregnancy Bill,” *British Medical Journal*, 1, no. 5539 (March 1967): 569–570.

62. DP Cocks (1966), “Termination of Pregnancy Bill,” *British Medical Journal*, 2, no. 5528 (December 1966): 1531.

63. Joan Busfield (1998), “Restructuring Mental Health Services in Twentieth-Century Britain,” in *Cultures of Psychiatry and Mental Health Care in Postwar Britain and the Netherlands,* Marijke Gijswijt-Hofstra and Roy Porter, eds (Atlanta: Editions Rodopi, 1998), 9–28.

64. Boyle, *Rethinking,* 17–18.

65. Ibid,18.

66. David Garland (2003), “The Rise of Risk,” *Risk and Morality,* ed. Richard V. Ericson and Aaron Doyle (Toronto: University of Toronto Press, 2003), 61.

67. P Taylor-Gooby et al. (1999), “Risk and the Welfare State,” *The British Journal of Sociology*, 50, no. 2 (December 1999): 177, https://doi.org/10.1111/j.1468-4446.1999.00177.x.

68. John Keown (1988) , *“Abortion*;” S J Macintyre (1973), “The Medical Profession and the 1967 Abortion Act in Britain,” *Social Science and Medicine*, 7, no. 2 (February 1973): 128, https://doi.org/10.1016/0037-7856(73)90154-6x

69. John Keown (1988) , *“Abortion*.”

70. Michael Thomson (2013), “Abortion Law and Professional Boundaries,” *Social & Legal Studies*, 22, no. 2 (June 2013): 191–210.

71. Drew Halfmann (2003), “Historical Priorities,” 572; Barr, “Battle,” 342.

72. Anon (1966a), “Therapeutic Abortion: Report by BMA Special Committee,” *British Medical Journal*, 2 (July 1966), 40–44.

73. The Lancet (1966), “B.M.A.’s Views on Abortion,” *The Lancet,* 2 (July 1966): 45–46; John Keown (1988), “*Abortion*,” 90.

74. Anon (1966b), “The Royal Medico-Psychological Association’s Memorandum on Therapeutic Abortion,” *British Journal of Psychiatry*, 112, no. 491 (October 1966): 1071–1073, https://doi.org/10.1192/bjp.112.491.1071

75. Ibid, 1073.

76. Ibid, 1072.

77. Ibid.

78. John Keown (1988), “*Abortion*,” 91.

79. Anon (1966d), “Legalised Abortion: Report by the Council of the Royal College of Obstetricians and Gynaecologists,” *British Medical Journal,* 1, no. 5491 (April 1966): 850.

80. Ibid, 850.

81. Ibid.

82. MP Joyston-Bechal (1966), “The Problem,” 307.

83. Anon (1966d), “Legalised Abortion: Report,” 852.

84. Anon (1966c), “Medical Termination of Pregnancy Bill: Views of the British Medical Association and the Royal College of Obstetricians and Gynaecologists,” *British Medical Journal*, 2, no. 5530 (December 1966): 1649–1650.

85. Ibid, 1649–1650.

86. Ibid, 1650.

87. Ibid.

88. Keith Hindell and Madeleine Simms (1971) , *“Abortion Law Reformed”* (London: Peter Owen, 1971), 25.

89. Deb H L (1965), 30 November 1965, vol. 270, cc 1142.

90. Deb H L (1965), 30 November 1965, vol. 270, cc 1143.

91. Deb H L (1965) 30 November 1965, vol. 270, cc 1153–1154.

92. Deb H L (1965), 30 November 1965, vol. 270, cc 1153.

93. Deb H L (1965) 30 November 1965, vol. 270, cc 1150.

94. Deb H L (1965) 30 November 1965, vol. 270, cc 1160.

95. Deb H L (1965) 30 November 1965, vol. 270, cc 1161.

96. Deb H L (1965) 30 November 1965, vol. 270, cc 1165.

97. David Steel (1971), “Foreword”, In *Abortion Law Reformed,* ed. Madeleine Simms and Keith Hindell (London: Peter Owen, 1971), 8.

98. Ibid, 8.

99. Ellie Lee (2003) , *“Abortion, Motherhood and Mental Health*, 5–16.

100. MP Joyston-Bechal (1966), “The Problem,” 308.

101. EW Anderson (1966), “Psychiatric Indications for the Termination of Pregnancy,” *Journal of Psychosomatic Research*, 10, no. 1 (July 1966): 131, https://doi.org/10.1016/0022-3999(66)90147-4.

102. RG Priest (1981), “*Editorial*,” in Colin Brewer, Ed, *Psychiatric Problems in Women Part 2: Some Psychiatric Aspects of Abortion and Contraception* (London: SK&F Productions, 1981), 1.

103. RG Priest (1972), “The Impact of the Abortion Act: a Psychiatrist’s Observations,” *British Journal of Psychiatry*, 121, no. 562 (September 1972): 294, https://doi.org/10.1192/bjp.121.3.293

104. Ibid, 294.

105. R Illsley et al. (1976), “Psychosocial Aspects of Abortion: A Review of Issues and Needed Research,” *Bulletin of the World Health Organization*, 53, no. 1 (February 1976): 101.

106. Raymond Illsley (1973), “Foreword” in *Experience with Abortion: A Case Study of North-East Scotland,* ed. Gordon Horobin (Cambridge: Cambridge University Press, 1973), vii–viii.

107. Dugald Baird (1966), “Experience at Aberdeen,” *Abortion in Britain: Proceedings of a Conference Held by the Family Planning Association at the University of London Union on 22 April 1966* (London, 1966), 16.

108. David Cantor and Edmund Ramsden (2014), “Introduction,” *Stress, Shock, and Adaption in the Twentieth Century* (Rochester: University of Rochester Press, 2014), 1–18.

109. Ibid, 1–18.

110. Ibid, 6–7.

111. Mark Jackson (2013), “*The Age of Stress: Science and the Search for Stability”* (Oxford: Oxford University Press, 2013), 179–182.

112. Ibid, 180.

113. D Baird (1967), “Sterilisation and Therapeutic Abortion in Aberdeen,” *British Journal of Psychiatry* 113, no. 500 (July 1967): 704, https://doi.org/10.1192/bjp.113.500.701.

114. Ibid, 704.

115. Rhodri Hayward (2012), “The Invention of the Psychosocial: an Introduction,” *History of the Human Sciences*, 25, no. 5 (December 2012): 8.

116. PH Tooley (1969), “If all Abortions are Legal, which are Desirable?,” *The Abortion Act 1967: Proceedings of a Symposium Held by the Medical Protection Society, in Collaboration with the Royal College of General Practitioners, at the Royal College of Obstetricians and Gynaecologists, London, 7 February 1969* (London, 1969), 12.

117. Ibid, 12.

118. R Illsley et al. (1976), “Psychosocial Aspects,” 83–106.

119. Ibid, 85.

120. RF Tredgold (1966), “A Psychiatrist’s View,” 13–14.

121. For a study of the arguments around this, see Ellie Lee (2003), “*Abortion, Motherhood and Mental Health*.” For medical studies in the 1970s, see, among others, Norman A Todd (1972), “Follow-Up of Patients Recommended for Therapeutic Abortion,” *The British Journal of Psychiatry*, 120, no. 559 (June 1972): 645–6; Colin Brewer (1977), “Puerperal Psychosis: Post-Abortion Psychosis and its Implications for the Aetiology of Puerperal Psychosis,” *Psychiatric Bulletin*, 1, no. 5 (November 1977): 6, https://doi.org/10.1192/pb.1.5.6; B Lask (1975), “Short-Term Psychiatric Sequelae to Therapeutic Termination of Pregnancy,” *British Journal of Psychiatry*, 126, no. 2 (February 1975): 173–177, https://doi.org/10.1192/bjp.126.2.173.

122. P Diggory (1966), “A Gynaecologist’s Experience,” *Abortion in Britain: Proceedings of a Conference held by the Family Planning Association at the University of London Union on 22 April 1966* (London, 1966), 90.

123. P Diggory (1965), “Psychiatric Opinions,” *The Lancet*, 286, no. 7426 (December 1965): 1348.

124. S Brooke (2001), “New World”.

125. Dugald Baird (1966), “Experience at Aberdeen,” 20.

126. Ellie Lee (2003) , *“Abortion, Motherhood and Mental Health,”* 169–170.

127. Pearce, “Discussion,” 15.

128. See, for example, J Macnicol (1989), “Eugenics and the Campaign for Voluntary Sterilisation in Britain Between the Wars,” *Social History of Medicine*, 2, no. 2 (August 1989): 147–169, https://doi.org/10.1093/shm/2.2.147.

129. Deb H L, 1967 26 July 1967, vol. 285, cc 1012.

130. Deb H L, 1967 26 July 1967, vol. 285, cc 1012–1013.

131. W Lindesay Neustatter (1966), “Change of Outlook,” 23.

132. HL Deb, 30 November 1965, vol. 270, cc 1234.

133. Ben Clements and Clive D. Field (2018), “Abortion and Public Opinion in Great Britain: A 50 year Retrospective,” *Journal of Beliefs & Values,* 39, no. 4 (2018): 3, doi:10.1080/13617672.2018.1441351.

134. Ibid.

135. Ibid, 11.

136. For the wording of the other questions, see appendix of Jaqueline Scott (1998), “Generational Changes in Attitudes to Abortion: A Cross-National Comparison,” *European Sociological Review*, 14 no. 2 (June 1998): 177–190, https://doi.org/10.1093/oxfordjournals.esr.a018233.

137. Wellcome Archives, “SA/ALR/D.11, Box 54, Madeleine Simms, The Ethical Foundations of Psychiatric Opinion in Abortion,” 6–8.

138. Simms, “Ethical Foundations,” 7.

139. HL Deb, 30 November 1965, vol. 270, cc 1146–1147.

140. Sally Sheldon (1997), “*Beyond Control*,” 32–33.

141. Ibid, 35–45.

142. J L D Fairfield (1967), “Termination of Pregnancy Bill,” *British Medical Journal*, 1, no. 5533 (January 1967): 173.

143. Ibid.

144. Deb H L, 1965 Deb, 30 November 1965, vol. 270, cc 1209.

145. A R Kemp (1967), “Termination of Pregnancy Bill,” *British Medical Journal*, 1, no. 5535 (February 1967): 299.

146. HC Mclaren (1968), “Sequels of Unwanted Pregnancy,” *The Lancet*, 292, no. 7568 (September 1968): 632, https://doi.org/10.1016/S0140-6736(68)90714-9.

147. P D John (1973), “The Impact of the Abortion Act,” *British Journal of Psychiatry*, 122, no. 566 (January 1973): 115–116, https://doi.org/10.1192/bjp.122.1.115-a.

148. DP Cocks (1966), “Termination of Pregnancy Bill,” *British Medical Journal,* 2, no. 5528 (December 1966): 1531.

149. Irving Zola (1972), “Medicine as an Institution of Social Control,” *The Sociological Review,* 20, no. 4 (November 1972): 500, https://doi.org/10.1111/j.1467-954X.1972.tb00220.x.

150. Ellie Lee et al. (2010), “Risk, Health and Parenting Culture,” *Health, Risk & Society*, 12, no. 4 (2010): 293–300.

151. David Paintin (2002), “The Abortion Act 1967, ICBH Witness Seminar Programme,” Held 10 July 2001, Institute of Contemporary British History, 2002, 46.

152. Ellie Lee et al. (2018), “The 1967 Abortion Act Fifty Years On: Abortion, Medical Authority and the Law Revisited,” *Social Science and Medicine,* 212 (September 2018): 26, https://doi.org/10.1016/j.socscimed.2018.07.010.

153. Eleanor Attar Taylor (2017), “British Attitudes to Abortion,” NatCen. 3 August 2017, www.natcen.ac.uk/blog/british-attitudes-to-abortion.

154. Clare Murphy cited in Sally Howard (2017), “After 50 years of Legal Abortion in Great Britain, Calls Grow for Further Liberalisation,” *British Medical Journal*, 359 (November 2017), https://doi.org/10.1136/bmj.j5278.

155. Sally Sheldon (2016), “British Abortion Law: Speaking from the Past to Govern the Future,” *The Modern Law Review*, 79, no. 2 (March 2016): 285, https://doi.org/10.1111/1468-2230.12180.
